# Green Synthesis of Copper Oxide Nanoparticles Synthesized by Terminalia chebula Dried Fruit Extract: Characterization and Antibacterial Action

**DOI:** 10.7759/cureus.50142

**Published:** 2023-12-07

**Authors:** Tharani Munusamy, Rajeshkumar Shanmugam

**Affiliations:** 1 Department of Pharmacology, Saveetha Dental College and Hospital, Saveetha Institute of Medical and Technical Sciences, Chennai, IND

**Keywords:** ecofriendly, antibacterial agent, wound pathogens, copper oxide nanoparticles, terminalia chebula

## Abstract

Introduction: Copper oxide nanoparticles (CuONPs) have emerged as potential antibacterial agents. In this study, we aimed to synthesize CuONPs using *Terminalia chebula* (*T. chebula*)* *dried fruit extract and evaluate their antibacterial activity against specific wound pathogens. Our primary objective was to comprehensively characterize dried *T. chebula *fruit (TCF)-CuONPs and assess their antibacterial efficacy.

Methods: CuONPs were synthesized through a green synthesis approach employing *T. chebula* dried fruit extract. Structural and compositional characterization involved UV-visible spectroscopy, scanning electron microscopy (SEM), elemental dispersive X-ray analysis (EDX), and transmission electron microscopy (TEM). The antibacterial activity of CuONPs was assessed against *Staphylococcus aureus,* *Pseudomonas aeruginosa,* and *Escherichia coli* through various assays, including agar well diffusion, time-kill curve, protein leakage analysis, and antibiofilm assays.

Results: Characterization revealed a distinct absorption peak at 440 nm in UV-visible spectroscopy, spherical morphology under SEM, and the presence of copper in EDX analysis. TEM revealed nanoparticle dimensions of approximately 10-12 nm. In antibacterial assays, TCF-CuONPs displayed significant efficacy, with Pseudomonas aeruginosa exhibiting heightened susceptibility.

Conclusion: This study successfully synthesized eco-friendly copper oxide nanoparticles using *T. chebula *dried fruit extract and thoroughly characterized their structural and compositional attributes. CuONPs exhibited substantial antibacterial potency against specific wound pathogens, indicating their potential in wound management applications. These findings contribute to the development of sustainable antibacterial solutions with implications for healthcare and environmental sustainability. Further research can delve into the mechanisms and broader applications of CuONPs based on the specific experimental outcomes.

## Introduction

Nanotechnology, a groundbreaking field with applications spanning medicine, electronics, energy, and environmental science [1], holds immense promise in revolutionizing wound healing within the medical domain. The process of wound healing, a complex sequence involving inflammation, proliferation, and remodeling stages [2], often faces hurdles in traditional approaches due to factors such as infections, inadequate blood supply, and underlying health conditions. The distinctive properties of nanotechnology offer a potential solution to these challenges [3].

Among the myriad of nanomaterials, copper oxide nanoparticles (CuONPs) have emerged as a particularly promising candidate in the realm of wound healing [4,5]. Copper, an essential trace element, plays pivotal roles in physiological processes, including angiogenesis, skin regeneration, and immune response. CuONPs, with their unique physicochemical attributes, efficiently deliver copper ions to wound sites, thereby promoting wound healing. Additionally, these nanoparticles exhibit potent antimicrobial properties, bolstering their potential in preventing wound infections [6,7].

The emergence of green synthesis methods has garnered attention due to their environmentally friendly and sustainable nature. In contrast to conventional physical and chemical approaches, which often involve toxic chemicals and high energy consumption, green synthesis harnesses biological entities such as bacteria, plants, and fungi for nanoparticle synthesis [8]. This not only mitigates environmental impacts but also enhances nanoparticle biocompatibility, rendering them more suitable for medical applications [9].

*Terminalia chebula *(*T. chebula*), a fruit-bearing tree native to South Asia, also known as Haritaki, stands as a well-established medicinal plant within Ayurveda, the traditional Indian system of medicine. Laden with bioactive compounds like tannins, flavonoids, and phenolic compounds, *T. chebula* can serve as both stabilizing and reducing agents during nanoparticle synthesis [10]. Furthermore, this plant possesses diverse medicinal properties, including antioxidant, anti-inflammatory, antimicrobial, and wound-healing attributes, further enhancing the therapeutic potential of nanoparticles [11]. The integration of nanotechnology into wound healing, particularly through the eco-friendly synthesis of copper oxide nanoparticles utilizing *T. chebula*, offers a promising strategy to surmount the limitations of conventional wound healing approaches. The utilization of medicinal plants such as *T. chebula* not only enhances nanoparticle biocompatibility and therapeutic efficacy but also contributes to the sustainability of the synthesis process.

In this study, we employ *T. chebula* as both a reducing and capping agent in the green synthesis of copper oxide nanoparticles. Subsequently, we subject these nanoparticles to a comprehensive array of characterization techniques. Furthermore, we investigate the antibacterial mechanisms of these green-synthesized copper oxide nanoparticles against three distinct wound pathogens.

## Materials and methods

Materials

The dried fruits of *T. chebula* were sourced from an herbal drugstore situated in Poonamallee, India.

Preparation of dried fruit extract

To prepare the *T. chebula* extract, 1 gm of powdered dried fruit was accurately measured and added to a beaker filled with 100 mL of distilled water. Subsequently, the mixture was subjected to boiling using a heating mantle set at 70°C for a period of 15-20 minutes. Upon completion of the boiling process, the mixture was carefully filtered through Whatman No. 1 filter paper. The resulting filtered extract was then stored in a refrigerator, awaiting use in the synthesis of nanoparticles.

Green synthesis of dried *T. chebula* fruit (TCF)-CuONPs

To synthesize copper oxide nanoparticles, a 20 mM solution of copper sulfate was measured and dissolved in 60 mL of distilled water. Subsequently, 40 mL of filtered* T. chebula* extract was introduced into the solution. The resulting reaction mixture was placed on a magnetic stirrer operating at a constant speed of 750 rpm and left undisturbed for 24 hours. Throughout this period, the progress of the reaction was routinely monitored by measuring the UV-visible spectra at specific time intervals up to the full 24-hour duration. Upon the completion of the reaction, the solution underwent centrifugation at 8000 rpm for 10 minutes to effectively collect the nanoparticle pellet. The supernatant was then carefully decanted, and the acquired pellet was subjected to thorough washing with double-distilled water, followed by a wash with ethanol. This process ensured the purification of the copper oxide nanoparticles. Finally, the purified nanoparticles were collected and stored in airtight Eppendorf tubes, ready for subsequent characterization and future applications.

Characterization of green synthesized nanoparticles

For the morphological analysis, transmission electron microscopy (TEM) and scanning electron microscopy (SEM) (JEOL FE SEM IT-800, [JEOL Ltd., Tokyo, Japan]) were employed. The absorption characteristics of the synthesized nanoparticles were ascertained via the utilization of a double-beam UV-visible spectrophotometer (ESICO - model 3375 [Electronics India Ltd., Panchkula, India]) encompassing a wavelength range spanning from 350 nm to 550 nm. Furthermore, energy-dispersive X-ray analysis (EDX) was implemented to determine the elemental composition of the sample.

Biomedical applications

Antibacterial Activity

In alignment with the methodology employed by Agarwal et al. in their research in 2018 [12], the present study will assess the effectiveness of TCF-CuONPs based on the size of the inhibition zones observed in the agar well diffusion assay. This approach ensures consistency with established practices and draws on the insights provided by previous research work in evaluating the antibacterial action of nanoparticles.

Time-Kill Curve Assay

To assess the bactericidal properties of CuONPs, a time-kill curve was employed, following a methodology similar to the one described in the author's research work in 2023 [10]; their correlation with the growth patterns of the three above-mentioned wound pathogens was analyzed. This assay involved the cultivation of these pathogens in Mueller Hinton broth with varying concentrations of CuONPs (ranging from 100µg to 1000µg) while tracking their growth at specified time intervals.

To ensure consistent results, preliminary growth curves were established, ensuring that the pathogens reached from a stable early phase to mid-log bacterial phase after a five-hour pre-incubation period in antimicrobial-free Mueller Hinton Broth. A 0.5 McFarland inoculum of each pathogen was meticulously prepared in sterile phosphate-buffered saline (PBS) and derived from cultures grown on Mueller Hinton agar plates at 37°C for 18-20 hours. Subsequently, 30 µL of this inoculum was diluted in 15 mL of pre-warmed (37°C) antimicrobial-free Mueller Hinton broth, and 90 µL of this resultant mixture was carefully dispensed into each well of a 96-well enzyme-linked immunosorbent assay (ELISA) plate. To each well, 10 µL of TCF-CuONPs at five different concentrations were added, and an untreated control was also included in the assay for reference purposes.

Protein Leakage Analysis

Protein leakage analysis was conducted using the Bradford assay method, following a methodology akin to the one detailed in the author's research work in 2023 [10]. Three wound pathogens were exposed to various concentrations (100µg, 250 µg, 500µg, 750 µg, 1000µg) of copper oxide nanoparticles synthesized through green methods for durations spanning 24 to 48 hours. Following the designated treatment period, centrifugation was done at 6000 rpm for 15 minutes to segregate the supernatant phase, which was subsequently collected for further examination. Each sample, comprising 200µL of the supernatant, was placed into 96-well ELISA plates. Then, Bradford reagent (800µL) was introduced into each well, and the plates were incubated in darkness for a duration of 10 minutes. Amoxicillin served as the standard reference in this assay. Optical density (OD) measurements of the samples were recorded at 595 nm.

Antibiofilm Assay

The antibiofilm assay encompassed the assessment of biofilm eradication efficacy, following a methodology reported by Sasarom et al. in their research in 2023 [13]. Initially, biofilms were permitted to mature within a 96-well microtiter plate, following established protocols, over a 72-hour incubation period. Subsequent to incubation, the culture medium was withdrawn, and the wells were rinsed thrice with (PBS) phosphate-buffered saline to eliminate non-adherent cells. Following this, 300 µL of nanoparticle (NP) solutions were introduced into the wells. Upon a specified duration, the NP solutions were discarded, and the biofilms adhering to the plates were subjected to a triple PBS wash. The biofilms were meticulously scraped and homogenized, and the resulting samples were subjected to serial dilution in PBS. These dilutions were subsequently assessed using an ELISA reader, with measurements taken at 590 nm.

 *Statistical Analysis*

In this study, all experimental results were represented as mean values along with their corresponding standard deviations (SD), with each parameter being measured three times (n = 3). Data processing was conducted using GraphPad Prism version 8.0.1 (Graphpad Software Inc., La Jolla, CA) For the in vitro investigations, including the assessment of antibacterial activity, time-kill curve assay, protein leakage analysis, and antibiofilm assay, significant differences among the treatment means were determined using one-way analysis of variance (ANOVA) coupled with Tukey's post-hoc test (p < 0.05).

## Results

Visual observation

**Figure 1:**
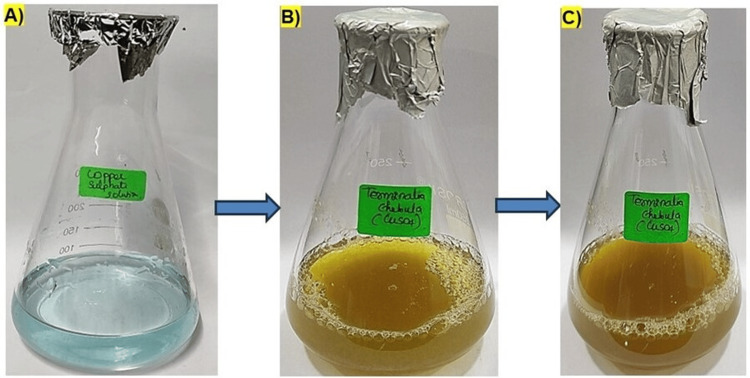
Visual observation image of T. chebula-mediated copper oxide nanoparticles (CuONPs). (A) Precursor solution; (B) T. chebula-mediated CuONPs - initial image; (C) final image T. chebula: Terminalia chebula; CuONPs: copper oxide nanoparticles​​​​​​

**Figure 2:**
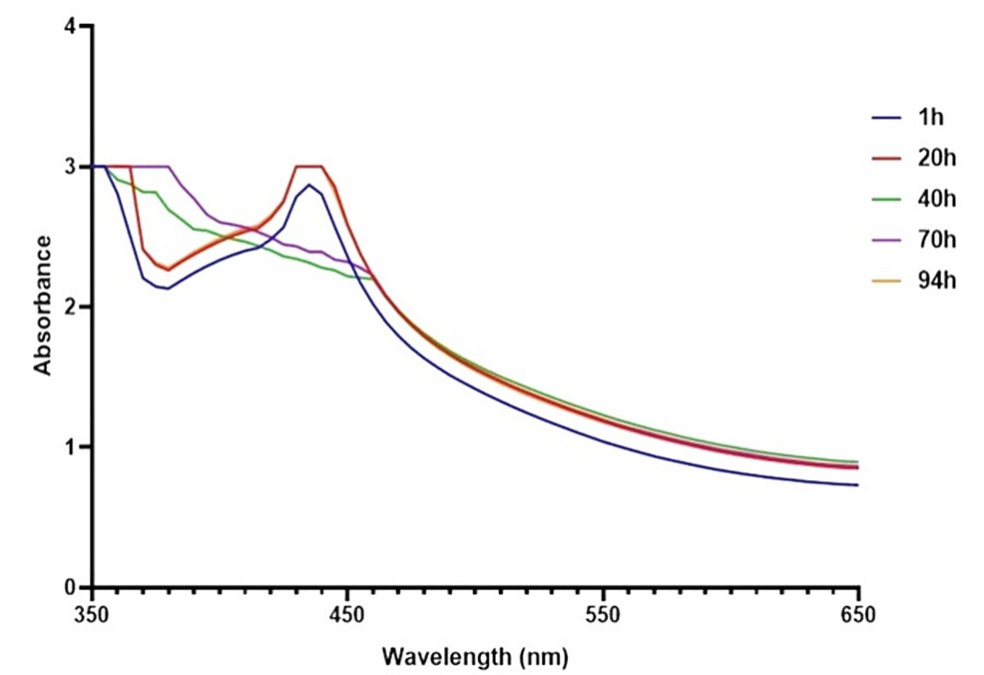
UV-visible spectra of T. chebula-mediated CuONPs T. chebula: Terminalia chebula; CuONPs: copper oxide nanoparticles

The synthesis of copper oxide nanoparticles (CuONPs) mediated by* T. chebula* extract was successfully carried out. When the precursor solution (Figure 1A) was mixed with *T. chebula* extract, *T. chebula-*mediated CuONPs were formed (Figure 1B). The initial image (Figure 1B) of the CuONPs showed a distinct golden brown color, indicating the presence of synthesized nanoparticles. Over the course of the reaction, the color of the solution gradually changed to dark brown. This transformation in color in Figure 1C signified the completion of the nanoparticle synthesis process. The visual observation of the CuONPs confirmed the successful reduction of copper ions using the *T. chebula* extract as a green and eco-friendly reducing agent which was depicted in Figure 2. The dark brown color of the final image (C) indicates the formation of stable CuONPs, and this transformation suggests the reduction of Cu(II) to Cu(I) during the synthesis process [13-14].

UV-visible spectrophotometry

UV-visible spectroscopy was performed to characterize the optical properties of *T. chebula*-mediated CuONPs. The analysis revealed a prominent absorption peak at a wavelength of 440 nm (Figure 2). The UV-visible spectrum displayed a strong absorption band centered at 440 nm, indicating the presence of CuONPs. The absorption peak corresponds to the electronic transitions within the CuO nanomaterials, specifically involving the excitation of electrons from the valence band to the conduction band. The observed absorption peak is consistent with the literature-reported optical properties of CuONPs. The location and intensity of the absorption peak provide valuable insights into the size, morphology, and composition of the synthesized CuONPs. The absorption maximum at 440 nm suggests the formation of small-sized CuONPs, as larger particles tend to exhibit absorption peaks at longer wavelengths. The efficient synthesis of CuONPs using the *T. chebula*-mediated approach is evident from the distinct absorption peak observed at 440 nm. The choice of* T. chebula* as a reducing and capping agent has proven successful in producing stable and well-defined CuONPs with desirable optical properties. Further characterization techniques, such as X-ray diffraction (XRD) and transmission electron microscopy (TEM), should be employed to confirm the crystalline nature, size, and shape of the synthesized CuONPs, providing a comprehensive understanding of their structural and optical characteristics. In conclusion, the UV-visible spectroscopy analysis of *T. chebula*-mediated CuONPs revealed a maximum absorption peak at 440 nm, indicating the successful synthesis of CuONPs. These findings support the potential use of *T. chebula *as a promising natural source for the fabrication of CuONPs with distinct optical properties.

Scanning electron microscope (SEM)

**Figure 3:**
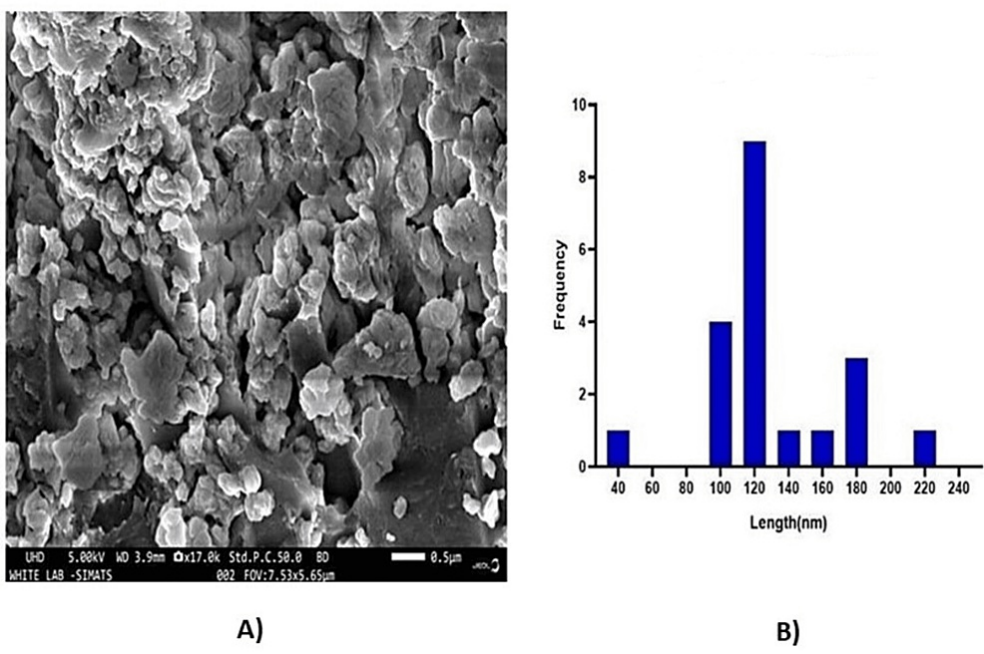
SEM image of T. chebula-mediated CuONPs. (A) SEM image; (B) histogram of CuONPs SEM: Scanning electron microscope; T. chebula: Terminalia chebula; CuONPs: copper oxide nanoparticles

The scanning electron microscopy (SEM) analysis of *T. chebula-*mediated CuONPs revealed their morphological characteristics, which can be seen in Figure 3. The nanoparticles exhibited a spherical shape and were observed to be aggregated. The size of the nanoparticles was determined to be approximately 120 nm. The SEM image showed that the CuONPs synthesized using *T. chebula* displayed a uniform spherical morphology. The nanoparticles appeared to be well-dispersed, forming aggregates of varying sizes. The aggregation of nanoparticles is a common phenomenon during the synthesis process, and it can be attributed to various factors such as van der Waals, electrostatic interactions, and solvent evaporation effects. The average size of the synthesized CuONPs was measured to be approximately 120 nm. The size determination was based on the analysis of multiple nanoparticles from the SEM image. It is important to note that the observed size may slightly vary due to the aggregation of nanoparticles, leading to overlapping or partial obscuration of individual particles.

Elemental dispersive analysis (EDX)

**Figure 4:**
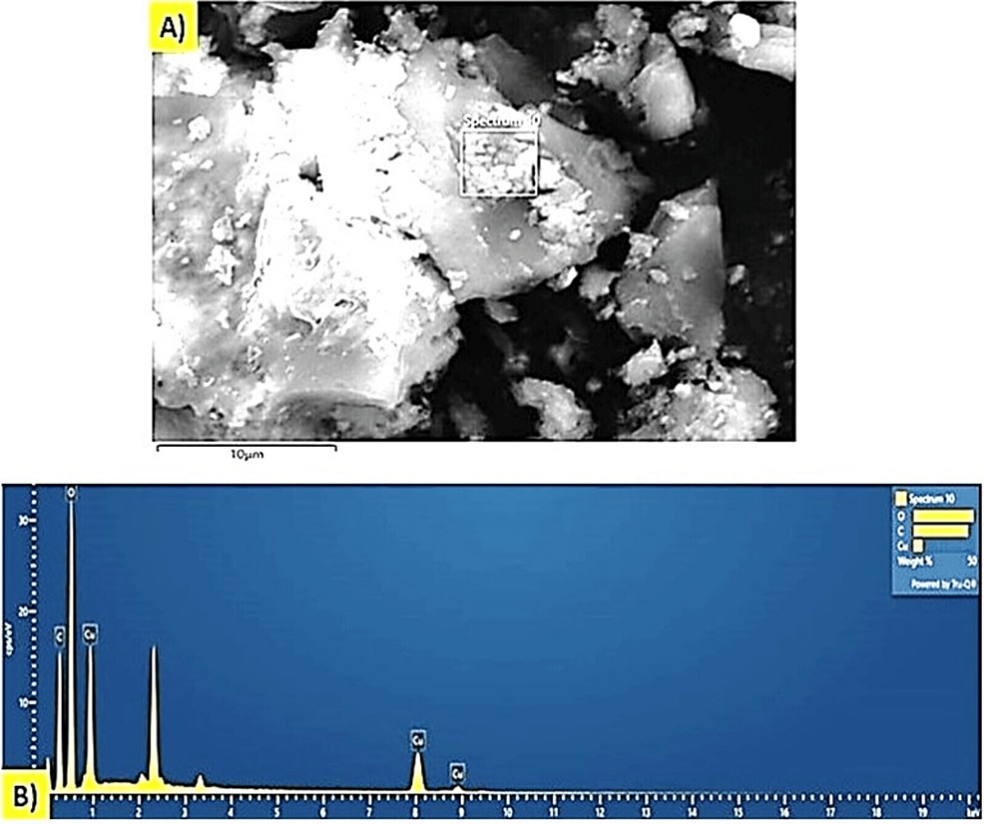
Elemental dispersive analysis of green synthesized CuONPs. (A) Spectrum image; (B) EDX spectra image CuONPs: copper oxide nanoparticles; EDX: energy-dispersive X-ray

Energy-dispersive X-ray spectroscopy (EDX) analysis was conducted to determine the elemental composition of *T. chebula*-mediated CuONPs. The EDX spectrum revealed the presence of copper (Cu), oxygen (O), and carbon (C) as the major elements in the synthesized nanoparticles (Figure 4). The EDX analysis confirmed the elemental composition of the CuO NPs, with copper accounting for approximately 17% of the composition, oxygen comprising around 35%, and carbon constituting approximately 15%. These results indicate the successful synthesis of CuONPs nanoparticles using *T. chebula* as the green reducing and capping agent.

Transmission electron microscopy (TEM)

**Figure 5:**
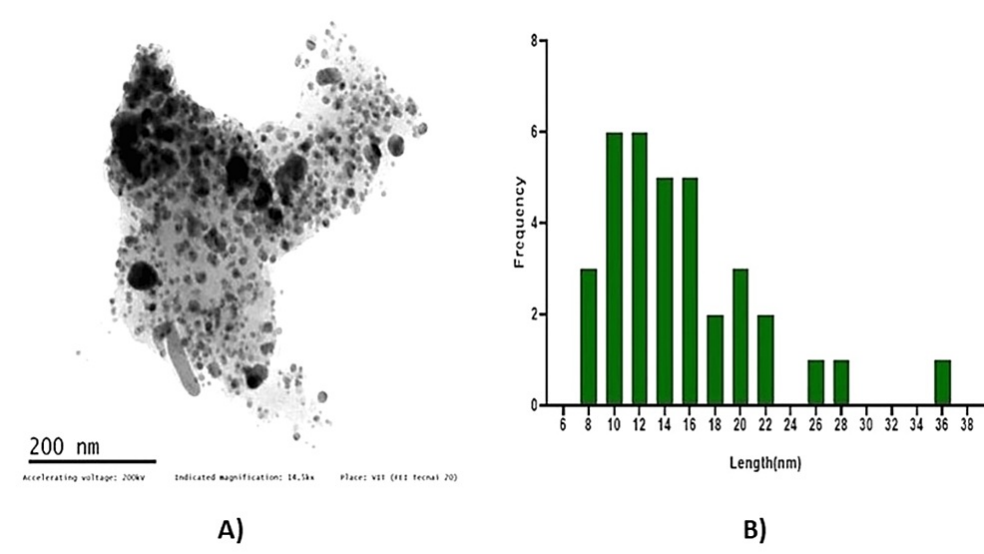
TEM image of green synthesized CuONPs. (A) TEM image; (B) histogram of CuONPs. TEM: Transmission electron microscopy; CuONPs: copper oxide nanoparticles

The transmission electron microscopy (TEM) analysis was performed to investigate the size distribution of copper nanoparticles synthesized using *T. chebula* extract. A representative TEM image of the synthesized copper nanoparticles is shown in Figure 5A. The TEM image revealed a homogenous distribution of nanoparticles throughout the sample. The analysis of the TEM image using ImageJ software (National Institutes of Health, Bethesda, MD) allowed for the determination of the average size of the copper nanoparticles. The average size was found to be approximately 10-12 nm, with a minimum size of 8 nm and a maximum size of 36 nm. The size distribution of the copper nanoparticles is shown in Figure 5B). The majority of the nanoparticles exhibited sizes within the range of 10-12 nm, as indicated by the peak in the size distribution curve. However, a small fraction of nanoparticles deviated from this range, with some reaching a size as small as 8 nm and others as large as 36 nm. The narrow size distribution observed in the TEM analysis indicates the effectiveness of *T. chebula* extract in controlling the growth of copper nanoparticles, resulting in a relatively uniform size range. The average size of 10-12 nm falls within the desired range for various applications, such as catalysis and biomedical research. These results demonstrate the potential of *T. chebula* extract as a green and efficient method for synthesizing copper nanoparticles with controlled sizes. The precise control over the size of copper nanoparticles opens up opportunities for tailoring their properties and applications in various fields.

Antibacterial activity:

Agar Well Diffusion Technique

**Figure 6:**
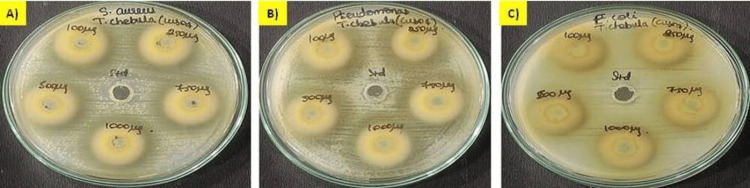
Antibacterial activity of T. chebula-mediated copper oxide nanoparticles against wound pathogens (A) S. aureus; (B) P. aeruginosa; (C) E. coli T. chebula: Terminalia chebula; CuONPs: copper oxide nanoparticles; S. aureus: Staphylococcus aureus; P. aeruginosa: Pseudomonas aeruginosa; E. coli: Escherichia coli

**Figure 7:**
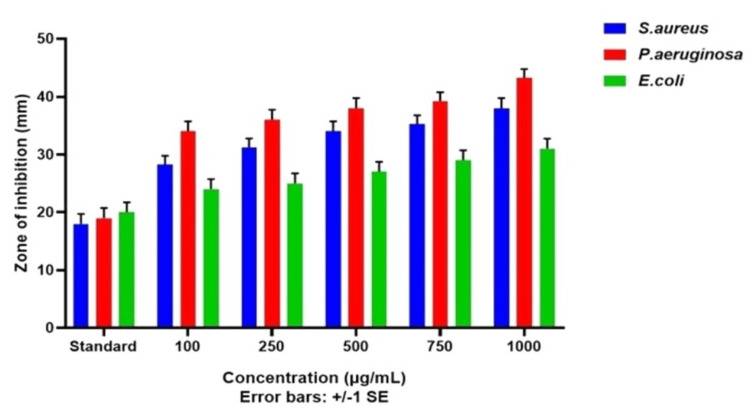
Graph demonstrating antibacterial activity of T. chebula-mediated CuONPs against wound pathogens T. chebula: Terminalia chebula; CuONPs: copper oxide nanoparticles; S. aureus: Staphylococcus aureus; P. aeruginosa: Pseudomonas aeruginosa; E. coli: Escherichia coli

The antibacterial activity of green synthesized CuONPs was tested using the agar well diffusion technique against three different wound pathogens, as depicted in Figures 6-7. In Figure 6, we present the results of the antibacterial activity of *T. chebula*-mediated CuONPs. This figure visually represents the data obtained from our experiments. Figure 7 provides a graphical representation of the antibacterial activity of *T. chebula* (CuONPs) against the tested wound pathogens. Against *Staphylococcus aureus *(*S. aureus*) the mean inhibition zone ranged from 25.67 ± 0.58 mm to 35.67 ± 0.58 mm, with the highest inhibition observed at 1000 µg/mL (15.67 ± 0.47 mm). The standard value exhibited an inhibition zone of 15.67 ± 0.47 mm. Against Pseudomonas, the mean inhibition zone ranged from 31 ± 0.82 mm to 40.67 ± 1.25 mm, with the highest inhibition observed at 1000 µg/mL (16 ± 0.82 mm). The standard value exhibited an inhibition zone of 16 ± 0.82 mm. Against *Escherichia coli *(*E. coli*), the mean inhibition zone ranged from 21.67 ± 1.25 mm to 28.67 ± 0.58 mm, with the highest inhibition observed at 750 µg/mL (17 ± 0.8 mm). The standard value exhibited an inhibition zone of 17 ± 0.8 mm. These findings suggest that CuONPs synthesized with *T. chebula* have significant antimicrobial potential against *S. aureus*, *Pseudomonas aeruginosa* (*P. aeruginosa*), and *E. coli*. The observed inhibition zones clearly demonstrate the nanoparticle’s ability to impede the growth of these bacteria. Notably, the highest inhibition zones were observed at higher nanoparticle concentrations, indicating a dose-dependent effect.

Time-kill curve assay

**Figure 8:**
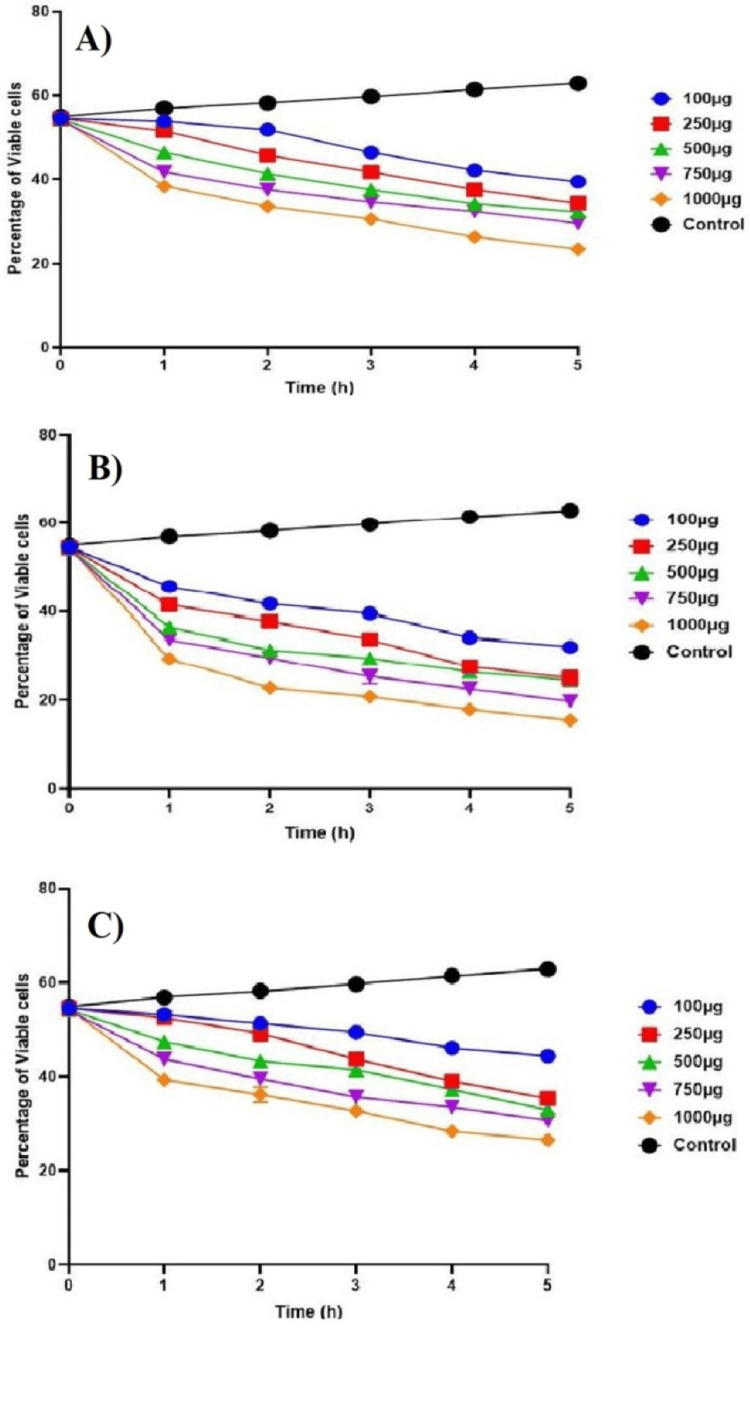
Time-kill curve assay of green synthesized CuONPs against wound pathogens (A) S. aureus; (B) P. aeruginosa; (C) E. coli CuONPs: copper oxide nanoparticles; S. aureus: Staphylococcus aureus; P. aeruginosa: Pseudomonas aeruginosa; E. coli: Escherichia coli

The time-kill curve assay of green-synthesized CuONPs against the three wound pathogens, *S. aureus, P. aeruginosa, *and* E. coli*, demonstrated concentration-dependent antibacterial effects when compared to the control group. At all concentrations of CuONPs (100µg, 250µg, 500µg, 750µg, and 1000µg), there was a noticeable reduction in *S. aureus* counts compared to the control group over the entire assay duration (Figure 8A). Notably, at the highest concentration (1000µg), a significant decline in *S. aureus* counts was observed within the first two hours, suggesting rapid bactericidal activity. In Figure 8B, similar to *S. aureus*, CuONPs displayed concentration-dependent bactericidal effects against *P. aeruginosa*. At all concentrations, CuONPs led to a substantial decrease in* P. aeruginosa* counts compared to the control group. Particularly at the highest concentration (1000µg), a significant reduction in *P. aeruginosa* counts occurred within the initial one hour, indicating potent antibacterial efficacy. In Figure 8C, CuONPs also exhibited concentration-dependent antibacterial effects against* E. coli*, although the reduction in bacterial counts was comparatively gradual. Even at the highest concentration (1000µg), the decline in *E. coli* counts was less pronounced, implying a lower susceptibility to CuONPs than the other two pathogens. Nonetheless, a noticeable reduction in *E. coli* counts was observed over time when compared to the control group. These findings collectively emphasize the concentration-dependent antibacterial properties of CuONPs against the tested wound pathogens. While *S. aureus* and *P. aeruginosa* displayed rapid susceptibility to CuONPs, *E. coli* exhibited a slower response. The comparison with the control group underscores the significant impact of CuONPs in reducing bacterial counts and highlights their potential as antibacterial agents for wound management.

Bradford assay

**Figure 9:**
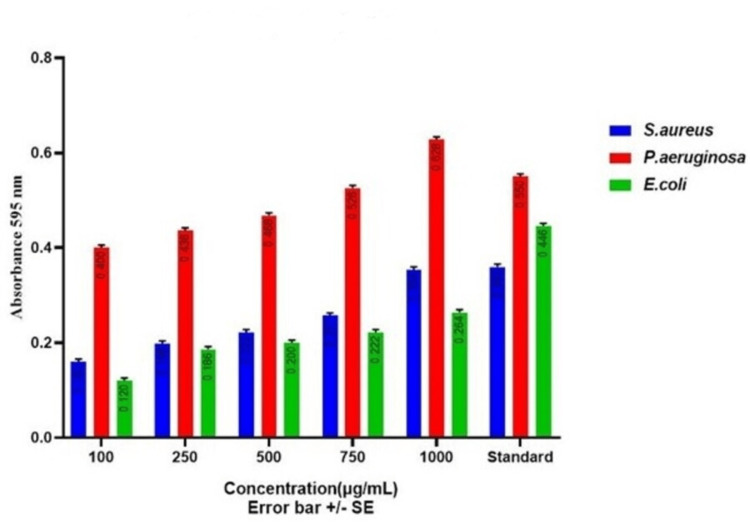
Bradford assay of green synthesized copper oxide nanoparticles against wound pathogens S. aureus: Staphylococcus aureus; P. aeruginosa: Pseudomonas aeruginosa; E. coli: Escherichia coli

Bradford or protein leakage assay was conducted to assess the impact of green-synthesized CuONPs on the integrity of the cell membranes of the wound pathogens, including *S. aureus, P. aeruginosa, *and* E. coli,* which was depicted in Figure 9.

Bovine serum albumin (BSA) served as the standard protein for comparison. CuONPs induced concentration-dependent protein leakage from *S. aureus* cells. As the CuONP concentration increased from 100µg to 1000µg, protein leakage gradually escalated. At 1000µg of CuONPs, the protein leakage reached 0.344, indicating a substantial impact on the cell membrane integrity of *S. aureus*. The protein leakage for other CuONP concentrations (100µg, 250µg, 500µg, and 750µg) showed similar concentration-dependent patterns when compared to the control group. Similar to *S. aureus*, CuONPs induced concentration-dependent protein leakage from* P. aeruginosa* cells. The protein leakage increased with higher CuONP concentrations. At 1000µg of CuONPs, the protein leakage reached 0.618, indicating a significant disruption of the cell membrane integrity of* P. aeruginosa*. The protein leakage for other CuONP concentrations (100µg, 250µg, 500µg, and 750µg) exhibited concentration-dependent patterns when compared to the control group. CuONPs also demonstrated concentration-dependent protein leakage from *E. coli* cells. The protein leakage increased gradually as the CuONP concentration increased. At 1000µg of CuONPs, the protein leakage reached 0.254, indicating a noticeable disruption of the cell membrane integrity of *E. coli*. The protein leakage for other CuONP concentrations (100µg, 250µg, 500µg, and 750µg) displayed concentration-dependent patterns when compared to the control group. These results collectively demonstrate that green-synthesized CuONPs induce concentration-dependent protein leakage from the tested wound pathogens. The effects on protein leakage were evident across all concentrations, with higher CuONP concentrations leading to more pronounced disruption of cell membrane integrity. This suggests that CuONPs have the potential to disrupt the cell membranes of these pathogens at various concentrations, which may contribute to their antibacterial mechanisms and potential applications in wound management. 

Antibiofilm activity

**Figure 10:**
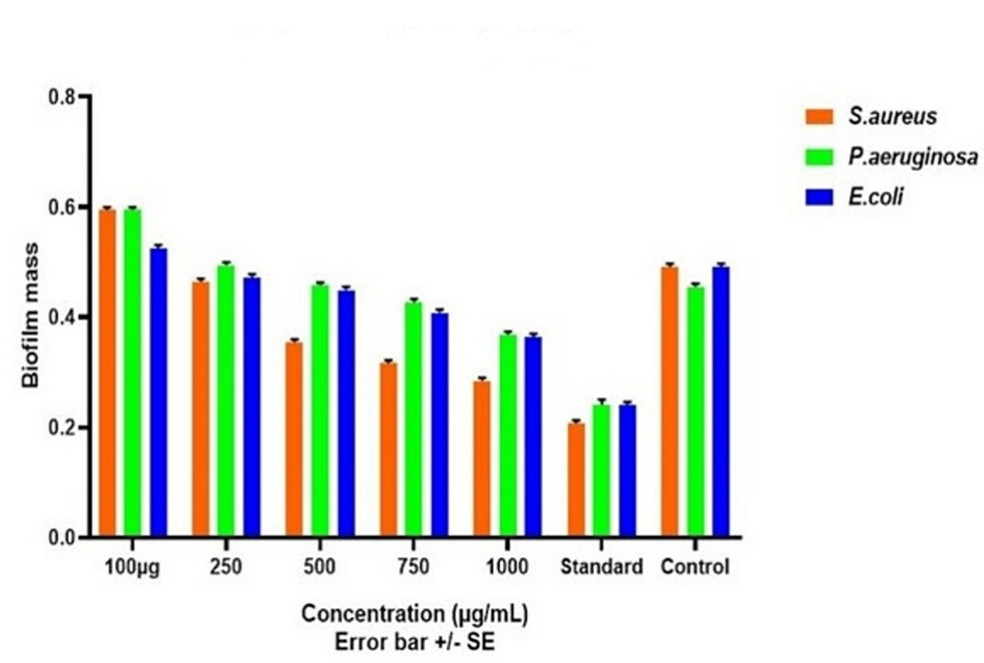
Antibiofilm activity of green synthesized copper oxide nanoparticles against wound pathogens S. aureus: Staphylococcus aureus; P. aeruginosa: Pseudomonas aeruginosa; E. coli: Escherichia coli

The antibiofilm activity of green-synthesized CuONPs against wound pathogens, including *S. aureus, P. aeruginosa, *and *E. coli *was evaluated by measuring absorbance values, which is depicted in Figure 10. CuONPs demonstrated concentration-dependent antibiofilm activity for all pathogens, with higher concentrations generally leading to more significant reductions in biofilm mass. Specifically, for *S. aureus*, absorbance values decreased from 0.584 at 100µg to 0.222 at 1000µg. For *P. aeruginosa*, absorbance values ranged from 0.582 at 100µg to 0.326 at 1000µg. Similarly, for *E. coli*, absorbance values decreased from 0.515 at 100µg to 0.352 at 1000µg. These findings highlight the potential of CuONPs as effective antibiofilm agents against wound pathogens and suggest their concentration-dependent efficacy in disrupting biofilms, which is crucial for wound management applications.

## Discussion

The successful synthesis of CuONPs mediated by* T. chebula* extract was evident through visual observation and various characterization techniques. The initial golden brown color (Figure 1C) of the CuONPs solution indicated the presence of synthesized nanoparticles, and the transformation to a dark brown color (Figure 1D) signified the completion of the synthesis process [15]. This visual observation confirmed the efficient reduction of copper ions using *T. chebula* extract as a green and eco-friendly reducing agent. UV-visible spectroscopy further characterized the optical properties of the CuONPs, revealing a prominent absorption peak at 440 nm, consistent with the literature-reported properties of CuONPs [16]. This peak provides valuable insights into the size and composition of the synthesized CuONPs, indicating their potential for various applications. SEM analysis displayed the spherical morphology of the CuONPs synthesized with *T. chebula* extract. Although some aggregation was observed, the average size of approximately 120 nm was determined, making them suitable for various applications. EDX analysis confirmed the elemental composition of the CuONPs, with copper, oxygen, and carbon as the major elements, validating the successful synthesis using *T. chebula* extract. TEM analysis provided a size distribution of the CuONPs with an average size of 10-12 nm, further demonstrating the control achieved over nanoparticle size [17].

The antibacterial activity of CuONPs against wound pathogens, including *S. aureus*, *P. aeruginosa*, and *E. coli*, was evident in both the agar well diffusion and time-kill curve assays. CuONPs exhibited concentration-dependent antibacterial effects, with higher concentrations leading to more pronounced reductions in bacterial counts [18,19]. Notably, *S. aureus* and* P. aeruginosa* displayed rapid susceptibility to CuONPs, while *E. coli *exhibited a slower response. These findings underscore the potential of CuONPs as antibacterial agents for wound management. Protein leakage analysis confirmed the disruption of cell membrane integrity in all tested wound pathogens by CuONPs. The effects were concentration-dependent, with higher CuONP concentrations inducing more significant protein leakage [20,21]. This disruption suggests a potential mechanism underlying the antibacterial activity of CuONPs. Additionally, CuONPs displayed concentration-dependent antibiofilm activity against all pathogens, with higher concentrations leading to more substantial reductions in biofilm mass. This concentration-dependent efficacy in disrupting biofilms is crucial for wound management applications [22,23]. Overall, the green synthesis of CuONPs using *T. chebula* extract demonstrated their potential for various applications, including wound management. The concentration-dependent antibacterial, protein leakage, and antibiofilm activities of CuONPs against wound pathogens highlight their promising role in addressing wound-related infections.

Limitations

The current study showcasing the synthesis and antibacterial potential of CuONPs using *T. chebula* extract presents valuable findings, yet it has limitations. It primarily focuses on a narrow range of wound pathogens in vitro, lacks in vivo experiments, overlooks safety concerns and biocompatibility, and does not address long-term stability or environmental impact. To be considered for clinical use, further research should encompass a broader spectrum of pathogens, in vivo studies, cytotoxicity assessments, and long-term stability, while also considering scalability, environmental impact, and regulatory approvals.

## Conclusions

In summary, the green synthesis of CuONPs using *T. chebula* fruit extract has resulted in nanoparticles exhibiting exceptional antibacterial properties. These properties hold significant potential for addressing the pressing issue of wound infections as well as mitigating the effects of oxidative stress and inflammation, both of which play crucial roles in the complex wound-healing process. The findings of this study provide a robust foundation for the ongoing exploration of CuONPs synthesized through green methods for therapeutic applications, with a particular focus on wound care and infection management. Leveraging their potent antibacterial attributes and excellent safety profile, these nanoparticles present a promising avenue for the development of innovative and effective treatments aimed at enhancing wound healing and reducing the burden of infections in the field of medical practice.
